# Achilles Tendon Surgical Repair Partially Restores Early Plantar Flexor Structure and Function in a Rat Model

**DOI:** 10.1002/jor.26041

**Published:** 2025-01-06

**Authors:** Ahmad Hammo, Liala Sofi, Lorraine A. T. Boakye, Josh R. Baxter

**Affiliations:** ^1^ Department of Orthopaedic Surgery Perelman School of Medicine, University of Pennsylvania Philadelphia Pennsylvania USA

**Keywords:** injury, mechanics, orthopedics, preclinical model

## Abstract

Achilles tendon ruptures significantly impair long‐term patient function, with two‐thirds of patients experiencing persistent functional deficits. Although nonsurgical treatment has gained popularity due to its perceived lower risk of complications, the specific effects of this approach on tendon healing, muscle function, and overall performance remain poorly understood. Directly comparing surgical and nonsurgical treatment options in a clinical population is challenging given the diverse nature of the patient population. Preclinical models are essential to isolate the mechanisms underlying these treatments, enabling a detailed examination of the structural and functional outcomes that are difficult to assess in human studies. Here, we surgically induced Achilles tendon ruptures in 20 adult male Sprague Dawley rats and repaired the rupture in half of these animals. Then, functional outcomes were assessed by measuring plantar flexor torque across the ankle's range of motion using a custom‐developed small animal dynamometer, and structural changes were evaluated through measurements of Achilles tendon elongation and plantar flexor muscle mass. We found that surgical treatment led to 11%–35% increased functional plantar flexor torque outcomes compared to nonsurgical treatment. Additionally, plantar flexor muscle mass decreased by 21% in nonsurgically treated animals compared to only 12% in the surgically treated group. Our results suggest that surgically repairing a tendon rupture restores plantar flexor function more effectively than nonsurgical treatment; however, persistent functional deficits in both groups indicate that enhanced rehabilitation strategies are necessary for full functional restoration.

## 1 Introduction

Over the past 30 years, Achilles tendon ruptures have increased 10‐fold, making them one of the most common tendon injuries among physically active individuals and the general population [1]. Achilles tendon rupture treatment often includes either surgical or nonsurgical approaches. Surgical treatment typically involves tendon suturing to restore anatomical alignment and function, while nonsurgical treatment relies on immobilization and natural healing without invasive repair. Although modern rehabilitative approaches have reduced re‐rupture rates from a staggering 40% to under 5% [2], functional impairments continue to impact most patients irrespective of surgical or nonsurgical treatments. Nearly two‐thirds of Achilles tendon rupture patients experience at least a 15% deficit in heel‐raise height a year after injury, regardless of the type of treatment received [3]. Many of these patients have compromised ankle biomechanics 6 years following the injury [4] and compensate for decreased ankle function by increasing knee joint work [5]. A recent meta‐analysis of 108 studies found that 20% of patients are not able to return to physical activities they enjoyed before the injury [6]. In addition to poor return to activity rates, the average patient who undergoes surgical repair of an Achilles tendon rupture requires 2 months before returning to work and at least 3–10 months before returning to play sports [7]. Despite these substantial burdens placed on the patient and society, optimal clinical treatment is still a hotly debated topic in the musculoskeletal community.

Recent trends [8, 9] and evidence [10, 11] suggest that nonsurgical treatment of acute Achilles tendon ruptures is a viable option. However, rigorous biomechanical studies that test the isolated effects of surgical and nonsurgical treatment on plantar flexor functional deficits are lacking. Our group's [12, 13] and others' [14, 15, 16] recent research have reported a link between plantar flexor structural changes and functional deficits following Achilles tendon rupture repair. Although these observational clinical studies test the functional implications of surgical versus nonsurgical treatment, they fall short in determining the underlying mechanisms of these functional deficits. A recent meta‐analysis [17] that investigated 822 patients over nine studies found that only three clinical reports quantified plantar flexor function using joint dynamometry and found small or no differences between surgical and nonsurgical treatment groups [2, 18, 19]. Unfortunately, these assessments do not fully characterize plantar flexor function, as they quantify instantaneous power or torque rather than plantar flexor work, which represents the ankle's kinetic capability to perform relevant tasks like jumping and heel raises. Therefore, there is a missing connection between biomechanical data and functional implications.

Preclinical animal models provide a powerful research platform to test clinically relevant questions. Recent small animal experiments suggest that nonsurgical treatment results in superior Achilles tendon biomechanics and healing post unilateral full‐thickness transection of Achilles tendon compared to surgical repair treatment in a rat model [20]. Unfortunately, there have not been any preclinical experiments that compare plantar flexor functional deficits between surgical and nonsurgical repair in a way that can be directly translated to clinical practice. Our group recently developed a small animal dynamometer that quantifies plantar flexor work and function in a rat model [21]. We tested the effects of nonsurgically repaired Achilles tendon ruptures in adult male rats and found that plantar flexor work deficits of 34% were similar in magnitude to plantar flexor work deficits of 38% we calculated in a cohort of patients treated nonsurgically [21]. This small animal dynamometer provides a translational functional assay to provide high‐quality evidence for clinical decision‐making.

This study aims to test the comparative outcomes of surgical versus nonsurgical treatment of acute Achilles tendon ruptures using an established rat model. We hypothesized that surgical repair of the ruptured tendon would yield enhanced plantar flexor function relative to nonsurgical treatment. We calculated plantar flexor torque and work across the ankle's range of motion to determine any functional changes. Alongside this, we measured tendon elongation and plantar flexor muscle changes to determine whether the observed functional deficits were explained by structural adaptations following the injury and its subsequent treatment.

## 2 Methods

We surgically induced an acute Achilles tendon rupture in 20 adult male outbred Sprague Dawley rats (weight: 437.9 g ± 43.3 g; obtained from Charles River) and randomly assigned them to either undergo surgical repair or nonsurgical repair treatments. We performed all injuries and surgical repairs on the right limb to maintain consistent surgical technique and used the uninjured contralateral limb as a control. The surgical procedures and immobilization protocols are previously described in greater detail [20] and were approved by our Institutional Animal Care and Use Committee (IACUC). In short, we made a small incision near the Achilles tendon while the animal was anesthetized using isoflurane (Isoflurane, Phoenix Pharmaceutical, St. Joseph, MO). Then, we resected the plantaris longus tendon and severed the mid‐portion of the Achilles tendon using the blunt end of an 11‐blade. Animals that were surgically repaired underwent the Urbaniak variant of the Kessler repair using 4‐0 braided polyester suture [20] (Tevdek, Teleflex, Wayne, PA). We closed the skin with an absorbable suture (Vicryl 9‐0, Ethicon, Raritan, NJ) and then immobilized the ankle in unloaded plantarflexion using a straight plastic splint that put the ankle near 90°. We used cotton and self‐adhesive wraps to hold the leg in that position and used bone cement (Poly(methyl methacrylate), Thermo Fisher Scientific, Waltham, MA) to prevent the animals from chewing off the brace. Then, all animals completed 2 weeks of joint immobilization in plantarflexion followed by 2 weeks of unrestricted cage activity to simulate the early rehabilitation stages in patients. This is consistent with previous studies indicating an optimal immobilization period of 2 weeks to restore tendon properties [20]. We held the post‐injury immobilization protocol constant between the two experimental groups to reflect the consistent use of rehabilitation in clinical populations. We checked all animals daily during the 2‐week immobilization period, and we changed casts in the event of swollen toes or every 7 days. After 2 weeks of immobilization in plantarflexion, we removed the braces and allowed the rats unrestricted cage activity for 2 weeks to simulate return to full weight‐bearing in patients. We housed all animals in a conventional facility with 12‐h light/dark cycles, continuous access to food and water, and a chew toy. Each cage housed two animals that received their surgery and immobilization at the same time. All animal handling and welfare maintenance was in accordance with guidelines set forth by our IACUC.

We then measured bilateral plantar flexor function using a custom‐designed small animal dynamometer 4 weeks posttreatment [21]. We implanted a pair of 24‐gauge electrical wires around the sciatic nerve to maximally stimulate the plantar flexor muscles. To do this, we anesthetized each animal using isoflurane and maintained body temperature using a circulating hot water pad during the sciatic nerve dissection and a heat lamp during the dynamometer testing. We shaved the hindlimbs of each animal and made a small incision on the lateral aspect of the thigh and then carefully separated the fascial layer covering vastus lateralis and biceps femoris muscles to create a small window using scissors. By expanding this window with the blunt ends of dissection scissors, we created a cavity and cleared the connective tissues from around the sciatic nerve and carefully placed the electrode wires around it. We then filled this cavity with warmed mineral oil, which acts as an insulator while preventing the nerve from dehydrating. We repeated this procedure on both legs so that we could analyze contralateral ankle function to use as “control” data for our statistical analyses. We tested bilateral plantar flexor function and structure at the 4‐week timepoint. We did this for three reasons: (1) it reduced the number of animals we needed in this study, (2) it allowed us to perform matched pair comparisons, and (3) we typically evaluate contralateral limb structure–function at the same clinical timepoint as the injured side testing. We stimulated maximal plantar flexor contractions across the ankle range of motion in 20° increments from 60° dorsiflexion to 60° plantarflexion with the knee held in 30° of flexion. We tested these seven ankle positions with 15 s of rest over three sets of contractions. We randomized the order of each ankle position within a given contraction set to minimize the effects of fatigue and testing sequence bias. We delivered 500‐ms wave stimulations (100 Hz, 100 µs pulse width, 1–3 mA) using a constant current stimulator (DS3, Digitimer, UK). We then calculated the peak plantar flexor torque generated at each ankle position and the plantar flexor work done across the range of motion by integrating the area under the torque–angle curve. We used the isometric contractions to test the isolated effects of injury on the force–length properties of the muscle.

After we completed the dynamometer testing, we euthanized the animals using carbon dioxide inhalation, fixed the hindlimbs in 4% formalin, and measured tendon elongation and muscle atrophy. We secured each limb with the ankle at 90° and the knee fully extended using 3D‐printed brackets. We measured Achilles tendon length and gastrocnemius muscle length using a pair of dial calipers with 0.1‐mm precision. We measured tendon length as the distance between the calcaneal insertion and the medial gastrocnemius junction. We measured muscle atrophy by dissecting away the Achilles tendon at the muscle–tendon junctions and weighing the plantar flexor muscles (the gastrocnemius heads and the soleus) using a milligram precision scale after blotting the muscle dry with a paper towel to remove excess moisture. We used Statistical Parametric Mapping (SPM) using the “spm1d” library in Python, version 3.9.14, to analyze differences in plantar flexor work and torque deficits across the full range of ankle motion. This approach was chosen over conventional *t* tests to account for the continuous nature of the data and to reduce the potential bias introduced by multiple comparisons. By using SPM, we were able to perform a mixed model analysis, collapsing the data into percentage change from the contralateral limb and directly comparing the surgical and nonsurgical approaches over the entire range of motion. This method allowed us to robustly evaluate functional differences between groups while considering joint angle as a variable.

We compared the contralateral limb (control) of non‐repair animals versus their injured side (non‐repair) and the contralateral limb of repair animals versus their injured side (repair). Each comparison utilized SPM to assess the functional impact of surgical and nonsurgical treatments on plantar flexor work and torque deficits. The SPM analysis generated *t* statistics and *p* values across the full range of ankle angles, providing a comprehensive view of functional outcomes.

We calculated plantar flexor work as the integral of the plantar flexor torque curve over the range of −60° to 60° using the trapezoidal rule (“np.trapz” function in Python), providing a measure of total ankle work (N mm). We performed secondary analyses to determine whether plantar flexor work, tendon elongation, and muscle atrophy differed between controls and nonsurgical and surgical treatment groups using one‐way analysis of variance (ANOVA) in GraphPad. We also performed matched pairs comparisons to determine whether the injury affected plantar flexor function and structure. These matched pair comparisons (Figure S1) are clinically relevant because clinical evaluations typically compare between a patient's injured and unaffected limb.

Structural deficits, characterized as tendon elongation and changes in muscle mass, were evaluated using paired *t* tests to compare the injured and contralateral limbs within each treatment group, and these analyses were performed using GraphPad Prism version 10.0.3. These structural assessments aimed to determine whether the observed functional deficits were associated with changes in muscle–tendon unit properties following injury and treatment.

## 3 Results

**Figure 1:**
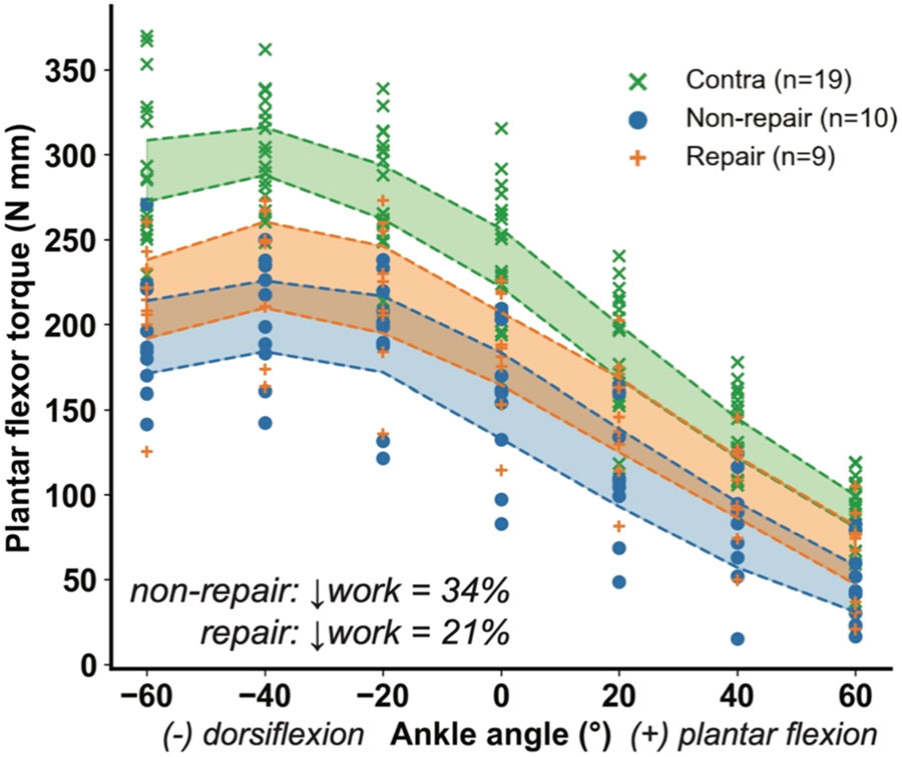
Plantar flexor active torques (N mm) at different ankle angles (°) during passive motion of the hindlimb, measured using a custom‐built dynamometer across treatment groups. The graph compares torque across the ankle range of motion in contralateral hindlimbs and non‐repair and repair groups following acute Achilles tendon rupture. Both non‐repair and repair treatments resulted in reduced torque compared to contralateral limbs. Shaded regions denote 95% confidence intervals.

**Figure 2:**
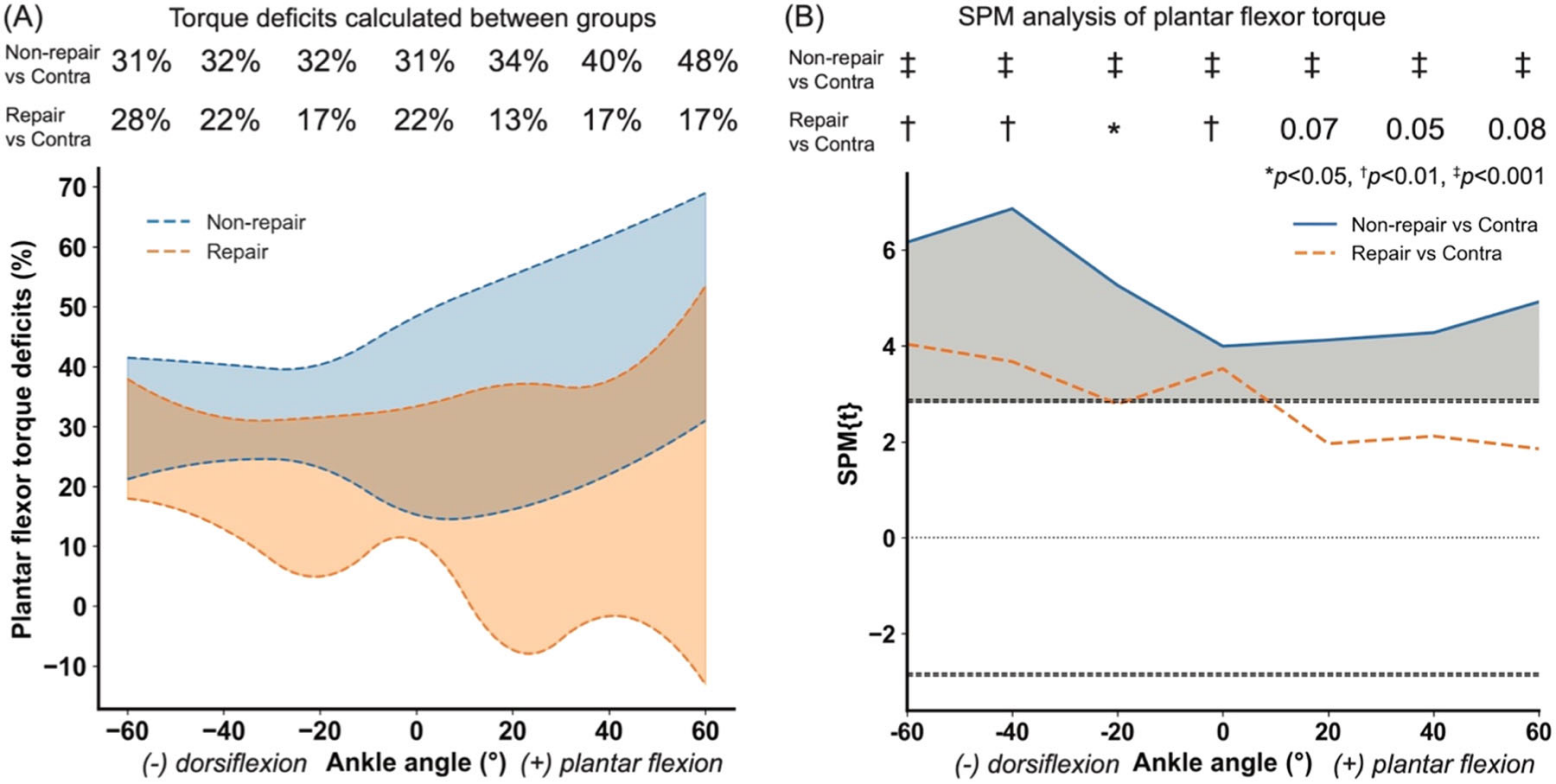
Plantar flexor torque deficits following treatment of Achilles tendon rupture. (A) 95% Confidence interval of plantar flexor torque deficits across ankle angles during passive motion for non‐repair and repair groups compared to their contralateral limbs. Percentages correspond to average torque deficit along condition. (B) SPM analysis of plantar flexor torque comparing non‐repair versus contralateral and repair versus contralateral limbs. The shaded region highlights areas of significant differences between groups, with the black dotted lines representing critical thresholds for statistical significance. Non‐repair animals show greater torque deficits, particularly in dorsiflexion and extreme plantar flexion, while repair animals exhibit lower deficits, especially in the plantar flexion range of motion. Statistical significance is marked across angles (*p < 0.05, †*p* < 0.01, ‡*p* < 0.001).

Plantar flexor torque was reduced across the ankle's range of motion following the Achilles tendon rupture and 4 weeks of recovery (Figures 1 and 2). Not surgically repairing the rupture led to a 34% reduction in plantar flexor work (*p* < 0.0001) while repairing the rupture led to a 21% reduction in plantar flexor work (*p* = 0.006) compared to the uninjured contralateral control limbs. Nonsurgical repair led to decreased plantar flexor torque across the range of motion ranging between 31% and 48% deficits compared to the contralateral limb (*p* < 0.001). Surgically repairing the tendon also resulted in plantar flexor torque deficits of 17%–28% (*p* < 0.05), but these deficits were only statistically significantly different in the dorsiflexion and neutral positions. These plantar flexor torque deficits—13%–17%—were smaller in the plantar flexed positions we tested and approached but did not reach statistical significance (0.05 < *p* < 0.08).

**Figure 3:**
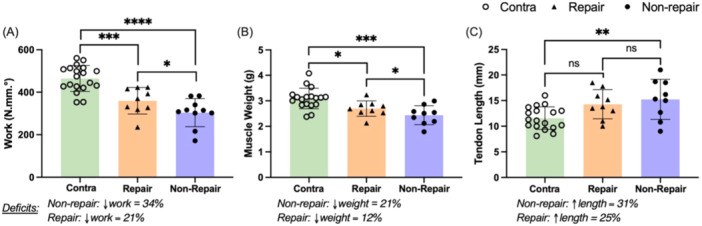
Comparative analysis of structural and functional outcomes following treatment of Achilles tendon rupture. (A) Plantar flexor work (N mm °), (B) muscle weight (g), and (C) tendon length (mm) in repair and non‐repair groups compared to the contralateral (control) limbs. Statistical significance is indicated by *p < 0.05, **p < 0.01, ***p < 0.001. Interval bars in (A–C) represent standard deviations from the mean. Average deficits between ipsilateral (injured) versus control limbs along conditions are displayed under each graph.

Surgically repairing the Achilles tendon rupture improved plantar flexor functional and structural outcomes compared to nonsurgical treatment (Figure 3). Plantar flexor work deficits were 62% greater in the nonsurgically repaired animals compared to the repaired group (*p* = 0.03, Figure 3A). The surgical repair (25% elongation) did not mediate tendon elongation compared to nonsurgical treatment (31% elongation). However, repairing the tendon reduced plantar flexor atrophy by nearly 50% (*p* = 0.04).

**Figure 4:**
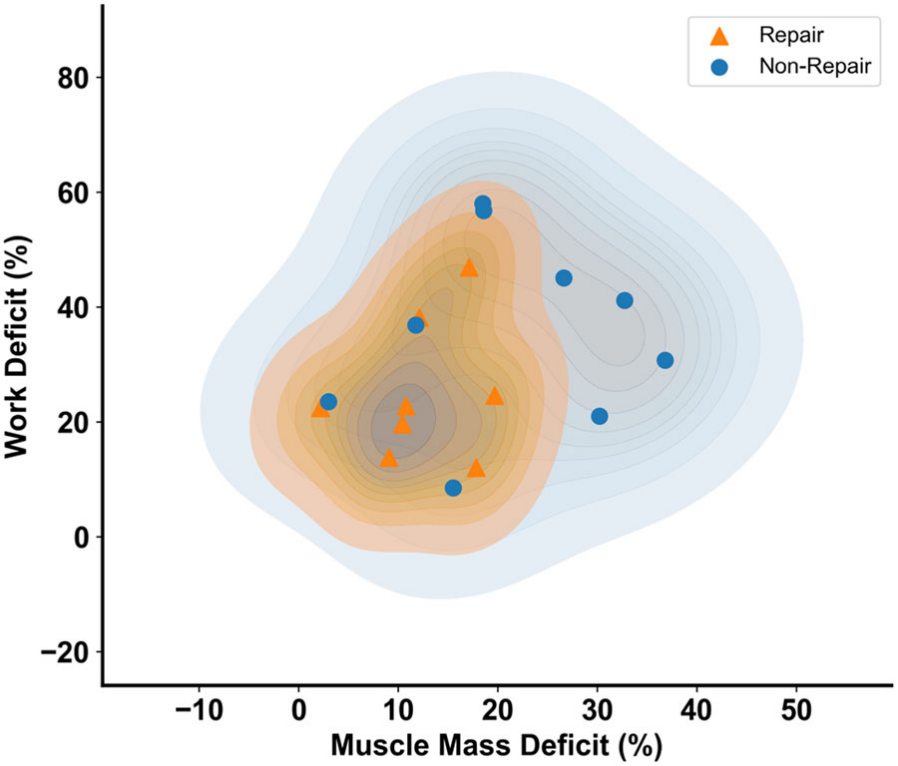
Comparative analysis of work and muscle mass deficits in repair and non‐repair groups following Achilles tendon rupture. The density plot displays the relationship between muscle mass deficits (%) and work deficits (%) for repair and non‐repair hindlimbs compared to their contralateral limbs. The shaded regions represent the density distribution of data points, with darker areas indicating higher concentrations of similar values. The orange‐shaded region represents the repair group, and the blue‐shaded region represents the non‐repair group.

Notably, surgically repairing the Achilles tendon led to a more consistent recovery profile than nonsurgical repair (Figure 4). The non‐repair group exhibited a greater standard deviation of ±10.9% in muscle mass deficits and ±16.5% in work deficits compared to the repair group, which had a ± 5.7% standard deviation in muscle mass deficits and ±11.9% in work deficits. This is represented by a tighter clustering of data points (Figure 4) in the surgically repaired group compared to the nonsurgically repaired group.

## 4 Discussion

Our study aimed to identify the isolated effects of surgically repairing an acute Achilles tendon rupture in a validated preclinical rat model. We quantified plantar flexor torque deficits across the range of ankle motion using a small animal dynamometer. Our findings support three clinically important conclusions: (1) an isolated Achilles tendon rupture leads to a compound muscle–tendon pathology that reduces plantar flexor functional capacity, (2) Achilles tendon rupture repair partly mitigates functional deficits and lowers the muscle–tendon structural variability compared to nonsurgical treatment in an established preclinical rat model, and (3) plantar flexor structure and function are not fully restored from surgically repairing the tendon.

Moreover, we demonstrated that although muscle–tendon structural and functional changes are present following both repair and non‐repair, nonsurgical treatment exhibits greater variability in results. We visualized the distribution of muscle mass deficits and plantar flexor work deficits and showed that surgically repairing the tendon leads to less uncertainty in structural and functional outcomes compared to nonsurgical repair. Non‐repair animals not only had higher average deficits but also demonstrated greater inconsistency in their recovery profiles compared to surgically treated animals.

The reduced variability in the surgical group suggests that tendon repair offers a more reliable and uniform response to treatment. This consistency is crucial, as it underscores the benefits of surgical repair not just in enhancing average functional outcomes but also in reducing the risk of unpredictable or suboptimal results. These findings emphasize the importance of surgical intervention in achieving predictable recovery trajectories, potentially guiding clinical decisions toward favoring surgical repair for more reliable restoration of function in Achilles tendon injuries.

Our findings provide new evidence that we expect will inform the debate on whether surgical treatment has practical benefits in restoring plantar flexor function. Recent trends [8, 9] and evidence [10, 11] suggest that surgically repairing the tendon has limited functional benefits while increasing complications associated with surgery. However, most evaluations of plantar flexor function rely on patient‐reported outcomes or isometric or isokinetic strength testing that evaluates ankle kinetics in the strongest part of the ankle range of motion. This is further compounded by younger and more active patients often opting for surgical repair while older and less active patients often opt for nonsurgical treatment.

Our findings show that surgically repairing the tendon mediates plantar flexor work deficits compared to nonsurgical treatment and may partly restore plantar flexor torque in plantar flexion—the range of motion most relevant for sport movements like running, hiking, and jumping. However, our findings suggest that surgically repairing the tendon does not fully restore plantar flexor function. We interpret this finding to mean that surgical treatment combined with standard immobilization is insufficient for promoting tendon healing and preserving muscle mass. Our future work is aimed at addressing the rehabilitation loading period to maximize functional and structural outcomes. Given that we used isometric contractions to test the isolated effects on injury on the force–length properties of muscles, our future work is also focused on evaluating movement‐specific rates of plantar flexion rotation.

Plantar flexor functional deficits should be considered a primary outcome measurement when designing preclinical and clinical research aimed at improving patient care. Both patient observational studies [13, 15, 22] and computational experiments [12, 23] suggest that tendon elongation and muscle structure are the primary drivers of plantar flexor functional deficits. Therefore, we suggest that future avenues of investigation focus on preventing tendon elongation and muscle atrophy while the tendon heals into a functional tissue. A recent study [24] found that patients had compromised plantar flexor function throughout the ankle range of motion despite not having strength deficits.

Our study findings provide new evidence that challenges the recent shift toward consideration of nonsurgical treatment of acute Achilles tendon ruptures—especially in the context of high‐demand individuals who aim to quickly return to a high level of activity. However, our study has several limitations that must be considered when considering our findings. The translational impact of our findings should be carefully considered within the context of our choice of animal model system and functional assay. Sprague Dawley rats have been used extensively as the preferred small animal model to investigate Achilles tendon healing [20, 25, 26]. This Achilles tendon rupture model required a surgical incision to transect the tendon. This poses several potential confounders: (1) opening the leg likely promotes an increased inflammatory response compared to spontaneous Achilles tendon ruptures, (2) nonsurgically repaired rats experienced this open approach that does not directly mimic the typical treatment in patients, and (3) we were unable to perform a percutaneous repair due to the small size and lack of precision instruments but the clinical evidence does not show clear advantages for improving patient function based on different surgical approaches. Despite these limitations, our recent comparison between rats and patients treated nonsurgically found similar plantar flexor functional deficits suggesting that our model closely mimics natural human injury [21]. Further, ankle dynamometry is a constrained activity compared to more functional activities like walking, running, or jumping. However, an earlier report [12] found that tasks that require doing positive plantar flexor work like the heel raise are more descriptive of functional deficits than energy‐return activities like walking [27]. We tested the effects of surgical and nonsurgical treatment on male rats because these injuries occur up to nine times more frequently in male patients [26]. However, our future work is focused on testing the effects of sex as a biological variable. Also, we tested plantar flexor function 4 weeks after the injury where the animals underwent 2 weeks of immobilization and 2 weeks of cage activity. Based on the rapid growth rate of rats compared to humans [28] and prior preclinical reports [20, 25], we approximated these 4 weeks to be equivalent to a typical 20‐week follow‐up visit for patients. Our future work will test functional outcomes at longer follow‐up visits in both patients and rats.

In conclusion, we tested the isolated effects of surgical and nonsurgical treatment of acute Achilles tendon rupture on plantar flexor functional outcomes. We found that surgically repairing the tendon leads to more consistent outcomes compared to nonsurgical treatment. However, our findings suggest that surgically repairing the tendon does not fully restore plantar flexor function, highlighting the need for further research focused on improved surgical treatment and optimized physical rehabilitation protocols.

## Author Contributions

J.R.B. initiated the project. A.H., L.S., and J.R.B. ran experiments and acquired data. A.H. and J.R.B. processed and analyzed the data. A.H., L.B., and J.R.B. wrote the paper.

## Conflicts of Interest

Josh R. Baxter serves on the ORS Tendon Section and JOR Editorial Board. The other authors declare no conflicts of interest.

## Supporting information


**Figure S1.** Comparative analysis of structural and functional outcomes following treatment of Achilles tendon rupture. (A) tendon length (mm), (B) muscle weight (g), (C) plantar flexor work (N mm °) in repair and non‐repair groups compared to their contralateral controls. Statistical significance is indicated by *p < 0.05, **p < 0.01, ***p < 0.001. Interval bars in (A–C) represent standard deviations from the mean. Average deficits between ipsilateral (injured) versus contralateral limbs are displayed under each graph.
