# Supplementary material for: Achilles Tendon Surgical Repair Partially Restores Early Plantar Flexor Structure and Function in a Rat Model
Source: J Orthop Res. 2025 Jan 6;43(4):739–45. doi: 10.1002/jor.26041 (PMC11898160; doi:10.1002/jor.26041)

**Supplemental Figures**

**Supplemental Figure 1.** Comparative analysis of structural and functional outcomes following treatment of Achilles tendon rupture. **(A**) tendon length (millimeters), **(B)** muscle weight (grams), **(C)** plantar flexor work (N.mm.°) in repair and non-repair groups compared to their contralateral controls. Statistical significance is indicated by *p<0.05, **p<0.01, ***p<0.001. Interval bars in panels A-C represent standard deviations from the mean. Average deficits between ipsilateral (injured) versus contralateral limbs are displayed under each graph.


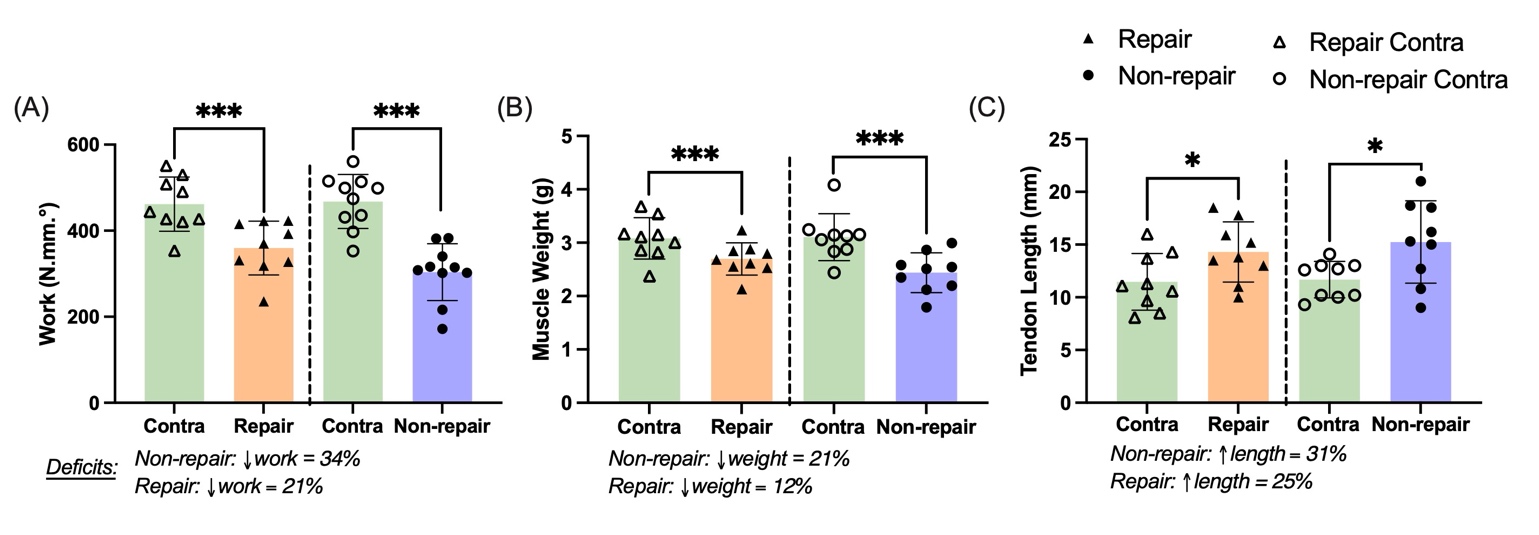

Supplement: Supplementary file 1 — Figure S1. Comparative analysis of structural and functional outcomes following treatment of Achilles tendon rupture. (A) tendon length (mm), (B) muscle weight (g), (C) plantar flexor work (N mm °) in repair and non‐repair groups compared to their contralateral controls. Statistical significance is indicated by *p < 0.05, **p < 0.01, ***p < 0.001. Interval bars in (A–C) represent standard deviations from the mean. Average deficits between ipsilateral (injured) versus contralateral limbs are displayed under each graph. [file JOR-43-739-s001.docx]
